# Evaluation of *Dactylopius opuntiae* Extract for Xanthine Oxidase Inhibition and Serum Uric Acid Reduction in a Hyperuricemic Mouse Model

**DOI:** 10.3390/ph17121575

**Published:** 2024-11-23

**Authors:** Othoniel H. Aragon-Martinez, Marco M. González-Chávez, Othir G. Galicia-Cruz, Santiago de J. Méndez-Gallegos, Mario A. Isiordia-Espinoza, Flavio Martinez-Morales

**Affiliations:** 1 Laboratorio de Productos Naturales, Facultad de Ciencias Químicas, Universidad Autónoma de San Luis Potosí, San Luis Potosí 78210, San Luis Potosí, Mexico; 2 Departamento de Farmacología, Facultad de Medicina, Universidad Autónoma de San Luis Potosí, San Luis Potosí 78210, San Luis Potosí, Mexico; othir@uaslp.mx; 3 Colegio de Postgraduados, Campus San Luis Potosí, Posgrado en Innovación en Manejo de Recursos Naturales, Salinas de Hidalgo 78622, San Luis Potosí, Mexico; jmendez@colpos.mx; 4 Departamento de Clínicas, Instituto de Investigación en Ciencias Médicas, División de Ciencias Biomédicas, Centro Universitario de los Altos, Universidad de Guadalajara, Tepatitlán de Morelos 47620, Jalisco, Mexico; mario.isiordia162@yahoo.com; 5 Independent Researcher, San Luis Potosí 78210, San Luis Potosí, Mexico; martinej@uaslp.mx

**Keywords:** wild cochineal, uric acid, kidney damage, antioxidant activity, carminic acid

## Abstract

**Background/Objectives**: Current urate-lowering therapies may cause serious side effects in patients. Thus, alternative treatments are needed to regulate uric acid (UA) levels in patients with hyperuricemia associated with kidney injury, and natural antioxidant sources have demonstrated utility in this field. For the first time, our study evaluated the effects of an extract of *Dactylopius opuntiae* insects on the levels of xanthine oxidase (XO) enzymes and synthetic free radicals in vitro and in vivo. **Methods**: Insects were bred and collected, and two different extracts (D1 and D2) were obtained. For both extracts, XO inhibition and radical scavenging assays were performed. Subsequently, serum purine levels and renal markers were quantified in male BALB/c mice who received a hyperuricemia induction using potassium oxonate, hypoxanthine, and gentamicin. **Results**: The D2 extract contained 18,037.7 µg/mL of carminic acid, inhibited 53.2% of XO activity at one concentration, and showed IC50 values of 18,207.8 and 5729.6 µg/mL against ABTS and DPPH radicals, respectively. D2 administration reduced serum UA and creatinine levels and prevented an increase in kidney weight and reduction in renal antioxidant capacity caused by hyperuricemia induction and allopurinol use in mice. Despite the satisfactory antioxidant results obtained in vitro, the D1 extract killed the animal models due to its citric acid content. **Conclusions**: The D2 insect extract can be used as an effective urate-lowering therapy when the increased level of serum uric acid is due to kidney damage.

## 1. Introduction

Uric acid (UA) is the end product of the purine degradation pathway in humans. In this pathway, xanthine oxidase (XO) is the enzyme that transforms hypoxanthine and xanthine into UA. Hyperuricemia, which is the excess accumulation of UA in the blood, can be caused by the metabolic overproduction or reduced renal excretion of UA [1]. Two-thirds of the total UA elimination primarily occurs via excretion by the kidneys. Hyperuricemia and renal function have a complex relationship. As kidney function declines, uric acid accumulates; however, this response is tempered by other compensatory excretion pathways that become activated. Consequently, hyperuricemia can result from impaired kidney function; however, in the clinical setting, hyperuricemia commonly precedes the development of chronic kidney disease. In this way, hyperuricemia causes kidney injury through the deposition of monosodium urate crystals in renal structures and surrounding vascular capillaries, as well as through the intracellular activation of mechanisms related to impaired renal flow, oxidative stress induction, and ischemia mediated by the soluble UA fraction [2].

The two main effective strategies used to prevent or treat hyperuricemia in patients are the use of XO inhibitors, such as allopurinol (AL) and febuxostat, to decrease UA production, and the use of uricosuric agents (e.g., benzbromarone) to increase UA excretion. However, both classes of drugs may cause serious side effects. For example, with regard to AL, there have been reports of reactions involving eosinophilia and systemic symptoms, toxic epidermal necrolysis, and gastrointestinal toxicity, among others. Febuxostat can inhibit the excretion of uremic toxins from the intestine through the ABCG2 channel, resulting in an increased incidence of cardiovascular events due to uremic toxin accumulation in patients undergoing dialysis. Benzbromarone has been associated with hepatotoxicity in patients. Therefore, there is a need for effective therapies with fewer serious side effects to regulate serum UA levels in patients. To this end, some extracts from natural sources have antioxidant properties that may be useful against hyperuricemia [1,3,4,5].

*Dactylopius opuntiae*, known as wild cochineal, is an insect considered to be an invasive pest of Opuntia species. Adult females are a source of carminic acid (CA) [6], which has been described as a potential therapeutic agent for fructose-induced kidney injury because it restricts inflammation and reactive oxygen species production and reduces the progression to chronic kidney damage [7]. Additional biological actions have been described for CA, such as antioxidant abilities, the attenuation of fatty liver disease progression, and suppressive activity against tumors. Meanwhile, a CA-rich extract obtained from *D. opuntiae* females has been reported to have properties of interest to the food industry, such as antioxidant activity and biochemical actions against the discoloration and oxidation of proteins and lipids in refrigerated meat [6].

To the best of our knowledge, *D. opuntiae* extracts have not been evaluated against the XO enzyme in vitro or in vivo. Therefore, the present study aimed to evaluate the effects of a *D. opuntiae* aqueous extract on XO inhibition, serum purine levels, and renal antioxidant markers in mice with experimentally induced hyperuricemia.

## 2. Results

### 2.1. D1 and D2 Insect Extraction

The extract obtained from method 2 (D2) exhibited a higher concentration of CA and a higher recovery fraction than the extract obtained from method 1 (D1) (Table 1).

### 2.2. Inhibition of the XO Enzyme

**Figure 1:**
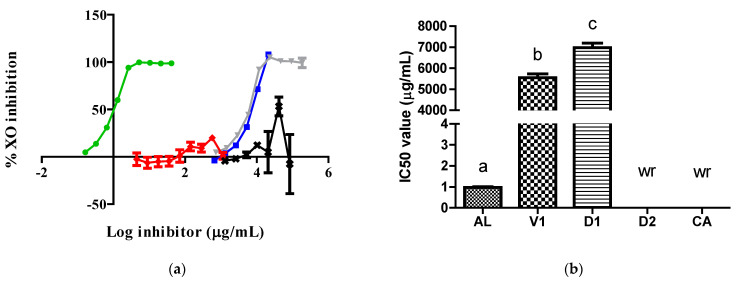
Activity of samples against the XO enzyme: (**a**) XO inhibition graph; (**b**) IC50 values calculated from curves. In (**b**), the lowercase letters a, b, and c above the bars indicate statistical differences (*p* < 0.0001). The green, red, gray, blue, and black lines represent the AL, CA, V1, D1, and D2 samples, respectively. wr, with no concentration–inhibition relationship. XO, xanthine oxidase; AL, allopurinol; V1, vehicle.

AL exhibited a higher inhibitory action, reflected by a lower IC50 value, against XO than the D1 and vehicle (V1) samples. Although the D2 and CA samples did not produce typical concentration–inhibition curves against XO, they showed inhibitory action values of 53.2 and 19.9% at 41,687.5 and 570.0 µg/mL, respectively (Figure 1).

### 2.3. Inhibition of Synthetic Radicals

**Figure 2:**
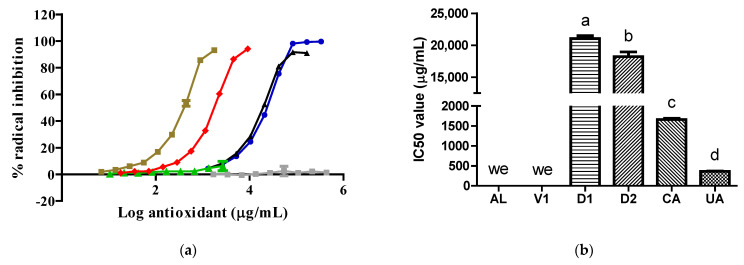
Activity of samples against the ABTS radical: (**a**) ABTS radical inhibition graph; (**b**) IC50 values calculated from curves. In (**b**), the lowercase letters a, b, c, and d above the bars indicate statistical differences (*p* < 0.0001). The green, red, gray, blue, black, and brown lines represent the AL, CA, V1, D1, D2, and UA samples, respectively. we, with no effect. ABTS, 2,2′-azino-bis 3-ethylbenzothiazoline-6-sulfonic acid; UA, uric acid. CA and V1 data were collected from a prior evaluation [6].

The UA samples exhibited a higher antioxidant activity, reflected by a lower IC50 value, against the 2,2′-azino-bis 3-ethylbenzothiazoline-6-sulfonic acid (ABTS) radical than the CA, D2, and D1 samples. The D2 samples showed superior antioxidant activity to the D1 samples. The V1 and AL samples did not inhibit the ABTS radical (Figure 2).

**Figure 3:**
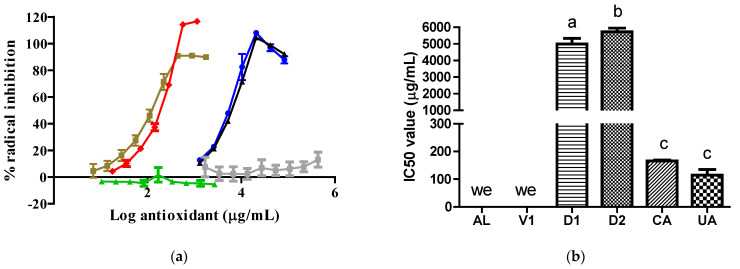
Activity of samples against the DPPH radical: (**a**) DPPH radical inhibition graph; (**b**) IC50 values calculated from curves. In (**b**), the lowercase letters a, b, and c above the bars indicate statistical differences (*p* < 0.0001). The green, red, gray, blue, black, and brown lines represent the AL, CA, V1, D1, D2, and UA samples, respectively. we, with no effect. DPPH, 2,2′-diphenyl-1-picrylhydrazyl. CA and V1 data were collected from a prior evaluation [6].

The UA and CA samples exhibited similar antioxidant activity against the 2,2′-diphenyl-1-picrylhydrazyl (DPPH) radical, reflected by similar IC50 values. This effect was superior to that exhibited by the D1 and D2 samples. The D2 samples had a lower inhibitory effect than the D1 samples. The V1 and AL samples did not inhibit the DPPH radical (Figure 3).

### 2.4. Effect of Experimental Samples in the Mouse Model

The administration of the V1 samples caused rapid death of the mice in the hyperuricemia state (H+V1). The time to death ranged between 1 and 4 min after the administration of the first dose. Moreover, the administration of the D1 samples caused the death of four of the six mice in the hyperuricemia state (H+D1) in a similar way to the V1 administration. The other groups did not show observable harmful effects over time.

The mice in the hyperuricemia and sodium carboxymethylcellulose administration (H+CMC) group exhibited higher serum UA levels than those in the control (Co) group. Under hyperuricemic conditions, the administration of D2 or AL reduced the serum UA levels in the mice to similar values to those observed in the Co group without statistical difference. The mice in the hyperuricemia and AL administration (H+AL) group exhibited higher serum levels of hypoxanthine and xanthine than those in the other groups. Higher hypoxanthine and xanthine levels were observed in the mice in the hyperuricemia and D2 administration (H+D2) group than in those in the Co and H+CMC groups but without statistical significance. Additionally, the serum creatinine level of the mice in the H+D2 group was inferior to that of the animals in the H group (Table 2).

**Figure 4:**
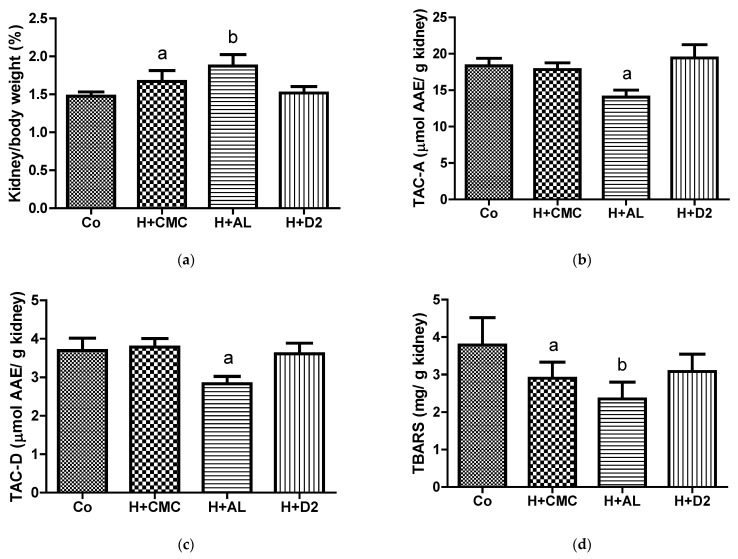
Effects of therapies in mouse kidneys: (**a**) kidney/body weight relationship; (**b**) ABTS radical inhibition by kidney supernatant; (**c**) DPPH radical inhibition by kidney supernatant; (**d**) TBARS amount found in kidney homogenates. For graph (**a**), ^a^ *p*-value of <0.05 versus Co and H+AL groups and ^b^ *p*-value of <0.0001 versus Co and H+D2 groups. For graphs (**b**,**c**), ^a^ *p*-value of <0.0001 versus Co, H+CMC, and H+D2 groups. For graph (**d**), ^a^ *p*-value of <0.05 versus the Co group and ^b^ *p*-value of 0.0009 versus the Co group. TAC-A, total antioxidant capacity against the ABTS radical; TAC-D, total antioxidant capacity against the DPPH radical.

As shown in Figure 4, hyperuricemia induction in the H+CMC group caused an increase in kidney weight and a reduction in the amount of renal thiobarbituric acid-reactive substances (TBARSs) compared with the Co group. Additionally, the administration of AL to this hyperuricemia model (the H+AL group) caused pronounced changes in kidney weight and TBARS levels, alongside a reduction in the renal antioxidant capacity, in comparison with the Co group. Finally, the H+D2 data showed that the administration of the D2 extract did not produce the increase in kidney weight and the reduction in the antioxidant capacity observed in the H+AL group.

## 3. Discussion

The CA contents in the D2 and D1 extracts were higher than those reported in a *D. opuntiae* extract sample obtained in a previous study using a procedure similar to that presented here (4497.1 ± 113.8 µg of CA/mL of M3 extract). They were also higher than the levels reported in *Dactylopius coccus* extract samples obtained via solid–liquid extractions with different solvents and temperatures (concentrations below 450 µg of CA/mL of extract) [6,8]. The present and previous extracts differed because of the amount of cochineal powder, type of cochineal, and volume of solvent used during the extraction process. Our data show that the extraction technique performed without citric acid was superior to that performed with citric acid (Table 1), despite previous data indicating that citric acid enhances CA extraction from a water matrix [8]. An explanation for this situation is that our procedure that used citric acid may have lost CA during sample processing due to having more preparative steps than the technique without citric acid. The comparison of the CA contents in the D2 and D1 extracts was possible since both preparations used the same proportion of the powdered sample per volume of solvent (0.334 g/mL), and the CA quantification was reported in mass of CA per mL of extract. Each proportion was obtained by dividing the sample grams by the solvent volume used (method 1: 3.34 g/10 mL and method 2: 6.68 g/20 mL). On the other hand, the amount of extract recovered depended on the volume of solvent used. In this way, method 1 used a lower volume than method 2, and consequently, method 1 obtained a lower amount of extract. Moreover, the amount of extract obtained from method 1 remained inferior to that obtained from method 2 if the amount of the two D1 extractions were combined. It is important to note that, although there are other methods that can be used to obtain CA-rich extracts from insect powder, they involve sophisticated equipment and high costs, such as those used for supercritical fluid extraction [8]. In contrast, solid–liquid extractions are easy and accessible procedures, as observed in the present methodology.

To the best of our knowledge, this is the first time that extracts obtained from insects belonging to the Dactylopius genus have been evaluated against XO. Our data show that the *D. opuntiae* aqueous extract had a very limited inhibitory effect on XO due to its key component, CA, having almost no effect on XO in comparison with AL (Figure 1). The inhibitory effect of the D1 extract on XO was due to its citric acid content, which was confirmed by the results obtained from the V1 samples. However, the D2 extract exhibited important action against the enzyme only at one concentration (Figure 1). The action of the D2 extract on XO was not typical of XO inhibitors. Possible reasons for this were the type of assay used, as it was a discontinuous assay, and the reaction time, which may have been insufficient to achieve an appropriate interaction between the inhibitor and the enzyme [9,10]. It is important to note that our finding that the V1 sample functioned as an XO inhibitor has not been reported in previous studies. Only one prior study mentioned that citrate preparations, which are conjugate bases of citric acid, improve serum UA levels, alongside AL, through an enhanced renal function in patients with hyperuricemia [11].

The antioxidant abilities of the D2 and D1 extracts (Figure 2 and Figure 3) were due to the content of CA, as this compound possesses free radical scavenging properties [7]. The antioxidant abilities found in the two extracts varied according to the CA content, type of radical, and CA solubility in the reaction medium. We report for the first time that the AL molecule did not possess antioxidant abilities by itself. In line with our result, previous data show that AL can inhibit free radical formation through its specific action against XO [12]. The V1 sample did not inhibit the two synthetic radicals tested because its major component, citric acid, did not possess antioxidant abilities [13]. Under the present experimental conditions, UA exhibited only antioxidant abilities without prooxidant actions [14].

An unexpected result was the death of mice due to the administration of V1 and D1 samples since both samples contain citric acid, a substance approved by the U.S. Food and Drug Administration as a food ingredient and widely used for human consumption [15]. The V1 administration involved a dose of citric acid/citrate at 3.98 g/kg bw, which is less than the no-observed-adverse-effect level (NOAEL) reported for citric acid in mice (7.5 g/kg bw/day). The dose was also less than a dose of citric acid used in albino mice (4 g/kg bw) in a lipopolysaccharide-induced endotoxemia model, where animal deaths were not reported [16,17]. A possible explanation for the animal deaths in this study is that citric acid administration worsened the kidney injury induced by gentamicin, resulting in acute hyperkalemia, which, in turn, produced fatal cardiac dysrhythmia. It is known that plasma citrate is filtered and reabsorbed at the glomerular and tubular levels in kidneys, respectively [18]. In the renal injury mice model, citrate was not eliminated from the body, resulting in its accumulation and action on the kidneys. It has been reported that a high dose of citric acid produces the disappearance of the basal membrane and fusion of tubules in the kidneys, which leads to a significant reduction in kidney function [19]. Hyperkalemia can be caused by renal failure and is associated with an increased risk of fatal cardiac arrhythmias [20]. Thus, we suggest that the death of mice was caused by the simultaneous administration of citric acid and gentamicin. As the D1 extract contained the same amount of citric acid/citrate as the V1 samples, D1 administration also killed the mice in the present model.

The H+CMC data confirmed that hyperuricemia was successfully induced under the animal protocol. A previous study demonstrated that hyperuricemia was not induced by the administration of potassium oxonate and hypoxanthine in BALB/c mice for 3 days. That data showed that the kidney had sufficient capacity to eliminate the excess serum UA caused by hypoxanthine administration and uricase inhibition through potassium oxonate [1]. In the present work, the addition of gentamicin to this previous animal protocol—potassium oxonate plus hypoxanthine for 3 days—allowed the serum UA to increase in mice. Thus, these results showed the causal relationship between renal insult by gentamicin and hyperuricemia and are in line with the literature showing the critical function of the kidney in the establishment of hyperuricemia [2]. Previous reports have confirmed that the reduction in the glomerular filtration rate (GFR) caused by gentamicin leads to the accumulation of metabolic products in blood, such as UA and creatinine [21,22,23,24]. The gentamicin-induced reduction in GFR is due to tubular, glomerular, and vascular effects. Moreover, kidney damage by gentamicin involves an inflammatory response with cell infiltration, activation of resident cells, increased cytokine production, and capillary hyperpermeability [21,22,23,24]. The use of gentamicin in our hyperuricemia mouse model increased kidney weight and serum UA levels. However, there was no statistically significant increase in the serum creatinine level following gentamicin administration because the duration of aminoglycoside exposure was short in comparison with that in previously published studies [21,22,23].

Our preclinical data demonstrate that the D2 insect extract can be used as a urate-lowering therapy if there is associated renal damage. Due to its antioxidant properties, it can protect against gentamicin-induced renal damage and lower serum UA levels. It is well established that co-treatment with antioxidants avoids the deleterious renal actions produced by gentamicin [24]. Currently, there are considerable studies in the literature that show the protective effects of nutrients with antioxidant activity on kidney function. Those studies show a strong association between the GFR and dietary total antioxidant capacity [25,26]. In our case, the D2 extract maintained low levels of serum creatinine and protected the kidney weight and antioxidant renal status in the mice subjected to hyperuricemia induction and AL administration (Table 2 and Figure 4). It is known that creatinine, a breakdown product of dietary meat and creatine phosphate, is mainly removed from the body by glomerular filtration in the kidneys. Meanwhile, UA, a purine breakdown metabolite, is mainly excreted by glomerular filtration and tubular secretion in the kidneys; however, UA also has a tubular reabsorption process [27,28]. Thus, both compounds, creatinine and UA, can suffer serum level changes if their excretory/reabsorption pathways are altered, such as by gentamicin exposure, as described in detail above.

Our results confirm that AL reduced serum UA levels by inhibiting the XO enzyme but increased renal damage in the mouse model, whereas the D2 extract reduced serum UA levels through protection against renal damage, as well as minor XO inhibition, as mentioned above. Thus, the D2 extract is a better intervention than AL if there is kidney damage. The H+AL data supported the in vivo inhibitory action of AL against the XO enzyme, while the H+D2 data did not show statistically significant changes in the xanthine or hypoxanthine levels (Table 2). This latter finding was in line with the poor activity of the D2 extract against the XO enzyme in vitro. Measurements of xanthine, hypoxanthine, and UA, which are involved in the purine catabolism pathway, are desirable to effectively interpret XO inhibition in vivo [1].

Our data show that AL had a deleterious effect on the kidneys during hyperuricemia induction in mice (Figure 4), which is consistent with previously reported data [4]. Our study shows that damage to the kidneys caused by hyperuricemia induction activated the antioxidant system, reflected by low TBARS values together with unchanged antioxidant levels. Moreover, this insult was exacerbated by AL administration, as a very low TBARS value was accompanied by a reduced antioxidant capacity in the kidneys (Figure 4). In some cases, inconsistent results between the TBARS and antioxidant levels were found. One explanation for these conflicting results is that antioxidant defense can be activated in response to an insult, which, in turn, prevents oxidative damage to lipids, and, thus, the generation of TBARSs is reduced while antioxidant levels remain unchanged [29,30]. Taking into account this explanation and the H+AL data, we suggest that the insult caused by AL was greater than the velocity of the response of the antioxidant renal system.

As this work involved in vitro and preclinical experiments, further evaluations, including clinical studies, are necessary to contribute to the evidence. This study had several limitations. First, histological examinations, monitoring of the urinary UA excretion, and transcriptomic, proteomic, or metabolomic tests were not performed, which would have increased the experimental evidence showing kidney dysfunction and recovery. Second, additional molecular mechanisms for the XO inhibition pathway and renal antioxidant status in mice were not explored. Third, the long-term effects of the extract were not monitored in the mice. Nevertheless, our study is the first to investigate the use of a cactus pest insect as a therapy to lower high urate levels linked to kidney damage. The use of an extract of *D. opuntiae* to treat patients, if confirmed by additional preclinical and clinical evaluations, would be highly feasible. It is an insect with a rapid reproduction rate, high resistance to environmental conditions, and a worldwide presence. There are large areas available for the plantation of its host plants (cactus) and practical techniques for its farming, and the procedure to obtain its extract is simple [31]. In the same manner as other drugs, the extract should be produced and manufactured under international and local regulations.

## 4. Materials and Methods

### 4.1. Insect Breeding

*D. opuntiae* [Cockerell] specimens and cladodes of *Opuntia ficus-indica* cv. Rojo pelón were obtained from an orchard belonging to the Colegio de Postgraduados, Campus San Luis, Salinas de Hidalgo, S.L.P., Mexico. Since 2011, Dactylopius specimens have been identified using microscopic features, and their reproduction has been maintained until now [32]. Cleaned and healed cladodes were placed in a container containing wet soil, and then they were exposed to *D. opuntiae* nymphs for 48 h. The infested cladodes were placed in a container covered with a fine mesh to produce adult female insects under controlled humidity (40%) and temperature (23.5 °C) conditions [33].

### 4.2. Cochineal Pigment Extraction

Adult female insects, alongside their wax, were collected, dried at 40 °C, and powdered with a blender. Subsequently, the insect powder was stored in polypropylene opaque containers at room temperature. Two conventional solid–liquid extraction methods were performed to obtain two cochineal extracts.

For the first method [6], a 433 mg/mL citric acid solution was prepared using drinking water, and then 3.34 g of the powdered insect sample was mixed with 10 mL of the citric acid solution (citric acid, Sigma-Aldrich, St. Louis, MO, USA). This mixture was incubated at 50 °C for 30 min, cooled at 4 °C for 14 min, and centrifugated at 1500 rpm for 10 min at room temperature. After centrifugation, three phases were identified, and the intermediate layer was collected. Then, 1.14 g of sodium carbonate (sodium carbonate, Sigma-Aldrich, St. Louis, MO, USA) was gradually added to the collected layer and maintained at room temperature until the formed foam disappeared completely. This mixture was centrifugated again, and two layers were produced. The lower layer was collected, weighed, and stored at −20 °C until use. In addition, the same procedure was applied for a citric acid solution without insect powder to obtain the V1 sample.

For the second method, 20 mL of drinking water was heated at 50 °C, and 6.68 g of the powered insect sample was mixed with the water for 5 min. This mixture was centrifugated at 1500 rpm for 10 min at room temperature. After centrifugation, four phases were identified, and the second layer from the top was collected, weighed, and stored at −20 °C until use.

### 4.3. CA Quantification

A CA quantification analysis was performed as described earlier [34] but with modifications. Calibrators containing CA (0.73–145 µg/mL) and fortified with AL (81.3 µg/mL) as an internal standard were prepared in deionized water. CA and AL reagents were acquired from Sigma-Aldrich (St. Louis, MO, USA). Subsequently, these calibrators were mixed with methanol (methanol, TEDIA, Fairfield, OH, USA) at a ratio of 1:1. Then, these mixtures were centrifugated (at 12,100 rpm for 10 min at 4 °C), and their supernatants were placed in vial inserts inside amber glass tubes (Agilent Technologies, Palo Alto, CA, USA) for a chromatographic analysis.

An 1100 series Agilent liquid chromatography system (Agilent Technologies, Palo Alto, CA, USA) consisting of a quaternary pump with a degasser, autosampler, thermostated column compartment, and diode-array detector, alongside Agilent ChemStation software for LC 3D systems (Rev. B.02.01-SR1), was used for measurements and data analyses. For chromatographic separation, a 250 mm long Syncronis C18 column (Thermo Fisher Scientific, Waltham, MA, USA) was used, with a 5 µm particle size and a 4.6 mm internal diameter. The column compartment was maintained at 30 °C during isocratic separation, and a mixture of acetic acid solution (3.49 g/L) and methanol at a ratio of 1:1 *v*/*v* was used as the mobile phase.

The preparation of extract samples included a dilution of 1:400 *v*/*v* with deionized water alongside fortification with AL. Each chromatographic peak in the extract samples was identified by its retention time and spectrum record, which were compared with those obtained from the calibrators. Monitoring was carried out at 274 and 254 nm for CA and AL alongside a drawing of the spectra from 190 to 400 nm. A flow of 1 mL/min for an 8 min run time and an injection volume of 10 µL for each sample were programmed in the chromatographic system for the separation. The CA concentration in each extracted sample was obtained by interpolating its response (ratio of the CA and AL peak areas) into the curve obtained with the calibrators.

### 4.4. XO Inhibition via Experimental Samples

This assay was performed as described previously [1]. In brief, working standards for the AL, CA, V1, D1, and D2 samples were prepared using drinking water (six to nine points per sample, n = 5). The assay was initiated with a mixture containing 100 µL of the test sample, 70 µL of 70 mM phosphate-buffered saline (PBS) solution (pH 7.5), and 60 µL of 0.37 U/mL XO, which was incubated at 37 °C for 30 min. After this incubation period, 120 µL of a 1 mM xanthine solution was added and mixed with the sample, and this subsequent mixture was incubated again at 37 °C for 30 min. After this second incubation period, the reaction was stopped by adding 50 µL of 1N HCl solution to the sample. Then, 200 µL of each sample was placed into a well of a 96-well plate, and the absorbance was read at 290 nm using a Cytation™ 3 microplate reader controlled using Gen5™ software version 2.06 (Biotek Instruments Inc., Winooski, VT, USA). For this assay, PBS (Na_2_HPO_4_ and KH_2_PO_4_), XO, xanthine, and HCl were obtained from Sigma-Aldrich (St. Louis, MO, USA). 

Using the data obtained from blank samples (samples processed as mentioned above but with the HCl solution added before XO), an interference-free absorbance was calculated for each tested sample. Each experimental result is expressed as the percentage of XO inhibition, which was calculated using the interference-free absorbance of the assay mixture, both with and without the test material.

### 4.5. Radical Inhibition via Test Samples

To evaluate the antioxidant capacity of the experimental samples, the DPPH and ABTS radicals were employed. These assays were carried out as described previously [35,36]. For both assays, working standards for the UA, AL, CA, V1, D1, and D2 samples were prepared using drinking water (seven to nine points per sample, n = 5). In this case, UA was used as a positive control, as it is a powerful antioxidant found in blood and renal tissues [14].

For the DPPH radical scavenging assay, a mixture of each sample (50 µL) and 450 µmol/L of DPPH solution (200 µL) was incubated at 30 °C for 20 min. After the incubation period, the mixture was centrifugated at 1500 rpm for 5 min at 15 °C, and the resultant supernatant (150 µL) was placed into a well of a 96-well plate to monitor the absorbance at 517 nm. Before the ABTS radical scavenging assay was started, the ABTS radical was produced by a reaction between ABTS diammonium salt and potassium persulfate in an aqueous medium. Subsequently, it was diluted with deionized water to obtain a 95.7 µmol/L ABTS radical solution. Then, a mixture of each sample (3 µL) and the diluted ABTS radical solution (300 µL) was incubated at 30 °C for 4 min. Finally, each mixture (150 µL) was placed into a well of a 96-well plate to monitor the absorbance at 730 nm. DPPH, ABTS diammonium salt, and potassium persulfate reagents were acquired from Sigma-Aldrich (St. Louis, MO, USA).

For both radical scavenging assays, the microplate reader and software version 2.06 described above were used to measure the absorbance units. The percentage of inhibition against the DPPH or ABTS radical was calculated using the absorbance values of the blank (drinking water) and experimental samples.

### 4.6. Mouse Model and Sampling

The procedure for the mouse model was carried out according to a prior study [1] but with modifications. Male BALB/c mice weighing between 15 and 20 g were kept at room temperature (21 ± 1 °C) and housed in acrylic cages (VWR International S. de R.L. de C.V., Tultitlán, Mexico) with free access to water and a standard diet under a 12 h dark and light cycle. The mice were housed under these laboratory conditions for one week before the experiment.

Aqueous solutions of 0.3% or 0.5% CMC (CMC, Sigma-Aldrich, St. Louis, MO, USA) were prepared using drinking water or sterile water, respectively. Hypoxanthine and AL (AL, Sigma-Aldrich, St. Louis, MO, USA) were suspended in 0.3% CMC aqueous solution, potassium oxonate (potassium oxonate, Sigma-Aldrich, St. Louis, MO, USA) was suspended in 0.5% CMC aqueous solution, and gentamicin (160 mg/2 mL of gentamicin, Antibióticos de México, Coyoacán, Mexico) was diluted at a ratio of 1:1 *v*/*v* with a physiological saline solution (solution of 0.9% sodium chloride, Laboratorios PISA, S.A. de C.V., Guadalajara, Mexico). The mice were randomly divided into the following six groups (n = 6 for each group): Co, H+CMC, H+AL, H+D1, H+D2, and H+V1 groups.

After 2 h of fasting, gentamicin (96 mg/kg bw, i.p.) alongside 0.3% CMC (9.2 mL/kg, p.o.), AL (2.5 mg/kg bw, p.o.), D1 (9.2 mL/kg bw, p.o.), D2 (9.2 mL/kg bw, p.o.), or V1 (9.2 mL/kg bw, p.o.) was administered once daily to the animals for three consecutive days.

On the third day, potassium oxonate (280 mg/kg bw, i.p.) was mixed with gentamicin for administration to the mice in the same injection. After one hour of the potassium oxonate/gentamicin injection and experimental oral therapy, a hypoxanthine suspension (268.0 mg/kg bw, p.o.) was administered to the mice. For the Co group, a physiological saline solution or a physiological saline solution plus 0.5% CMC was administered via an intraperitoneal injection, while 0.3% CMC was administered via the oral route for the same periods of time as the other groups.

One hour after hypoxanthine administration, the mice were anesthetized with a combination of ketamine and xylazine (100 and 20 mg/kg bw, respectively). Ketamine and xylazine were acquired from Laboratorios PISA S.A. de C.V. (Guadalajara, Mexico). Then, blood and kidneys were collected from the mice. The kidneys were placed in liquid nitrogen, while the blood was kept at 4 °C for 1 h. Subsequently, the blood samples were centrifuged at 5600 rpm for 10 min at 4 °C. The resultant serum samples were separated and frozen at −20 °C for further analysis. To continue the processing of the kidneys, they were thawed at 4 °C, weighed, and homogenized in 2 mL of a cold 70 mM PBS solution (pH 7.5). Subsequently, each homogenate was centrifugated at 11,200 rpm for 10 min at 4 °C. Finally, each supernatant was frozen at −20 °C for further analysis.

### 4.7. Serum UA, Xanthine, Hypoxanthine, and Creatinine Levels

A liquid chromatography method was used for the quantification of purines and creatinine, as described in a prior study [1]. In brief, the chromatographic conditions used were as follows: a column compartment maintained at 32 °C, a mobile phase consisting of a PBS solution (KH_2_PO_4_, Sigma-Aldrich, St. Louis, MO, USA) at pH 6.0 in an acetonitrile (acetonitrile, TEDIA, Fairfield, OH, USA) gradient, an injection sample volume of 20 µL, a flow rate of 1 mL/min, and multiple monitoring wavelengths (236, 248, 263, and 292 nm for creatinine, hypoxanthine, xanthine, and UA, respectively) together with spectrum drawing (ranging from 200 to 400 nm). The same apparatus, stationary phase, and software as described above were used for this chromatographic analysis. The compounds were identified by retention times and spectrum records. The compound concentration in the serum samples was calculated from the calibration curves obtained with the pure compounds. The results are expressed as μmol of compound/L of serum sample.

### 4.8. Antioxidant and Lipid Peroxidation Levels in Kidney Homogenate Supernatant

The ABTS and DPPH radical scavenging assays described above were employed. In the ABTS radical inhibition assay, the kidney supernatants were undiluted, and the blank sample was the 70 mM PBS solution (pH 7.5). For the DPPH radical inhibition assay, the kidney supernatants were diluted 1:1 with the 70 mM PBS solution (pH 7.5) before the reaction with the radical, and the blank sample was the PBS solution. The absorbance value obtained from each tissue supernatant was interpolated in a calibration curve prepared with ascorbic acid. Using the kidney weight and interpolated ascorbic acid concentration, the antioxidant ability of each sample is expressed in µmol of ascorbic acid equivalents (AAE)/g of the kidney.

To measure the lipid peroxidation in the samples, the TBARS assay was used. This assay was adapted from prior studies [37,38]. A sample of the kidney supernatant (200 µL), 20 µL of a 493 mg/mL trichloroacetic acid solution (trichloroacetic acid, Sigma-Aldrich, St. Louis, MO, USA), and 2.5 µL of a 1 mg/mL butylated hydroxytoluene solution (butylated hydroxytoluene, Sigma-Aldrich, St. Louis, MO, USA) were mixed for 1 min. This mixture was centrifuged at 4400 rpm for 10 min at 18 °C. A volume of the supernatant or malondialdehyde calibrator (100 µL) was mixed with 60 µL of a 0.8% thiobarbituric acid solution (thiobarbituric acid, Sigma-Aldrich, St. Louis, MO, USA). Then, this mixture was incubated at 75 °C for 30 min and kept at 4 °C for 3 min. A volume of the mixture (120 µL) was placed in 1 well of a 96-well plate to read the absorbance at 532 nm using the same microplate reader and software mentioned above. The TBARS value for each sample was calculated using the calibration curves of malondialdehyde. The malondialdehyde stock solution was obtained using 1,1,3,3-tetraethoxypropane reagent (Sigma-Aldrich, St. Louis, MO, USA) as described previously [37]. Considering the TBARS concentration, volume of the sample, and kidney weight, the results are expressed as mg TBARS/g of the kidney.

### 4.9. Data Analysis

Data are presented as the mean and standard deviation. Student’s *t*-test with Welch’s correction was used for the analysis of the CA content. A one-way ANOVA with Tukey’s post-test was used for the analyses of the data obtained from the XO inhibition experiments, antioxidant abilities in vitro, kidney weighing, and kidney homogenates. A *p*-value of <0.05 was considered statistically significant. The calculation of IC50 values and statistical analyses were performed using GraphPad Prism software version 5.01 (San Diego, CA, USA).

## 5. Conclusions

A *D. opuntiae* aqueous extract, free of citric acid and rich in the antioxidant compound CA, can be used as an alternative urate-lowering therapy to treat increased levels of serum uric acid due to kidney damage. The administration of this extract successfully reduced serum UA levels in mice by protecting against kidney oxidative impairment and, to a lesser extent, inhibiting XO.

## Figures and Tables

**Table 1:** Results of extraction procedures.

Parameter	D1	D2	*p*-Value
CA (µg/mL)	12,734.1 ± 1751.1	18,037.7 ± 1016.2	0.0002
Amount of extract (g)	2.11 ± 0.25	10.05 ± 0.58	<0.0001

Every value includes the mean ± standard deviation (n = 6). CA, carminic acid; D1, extract obtained from method 1; D2, extract obtained from method 2.

**Table 2:** Serum biochemical parameters evaluated in experimental animal groups.

Parameter	Co	H+CMC	H+AL	H+D2
UA (µmol/L)	123.3 ± 47.0	306.1 ± 127.4 ^a^	29.7 ± 12.1	145.0 ± 24.1
Hypoxanthine (µmol/L)	3.5 ± 2.0	4.3 ± 0.9	72.4 ± 49.3 ^b^	12.5 ± 5.2
Xanthine (µmol/L)	2.0 ± 3.2	4.5 ± 2.2	45.7 ± 28.1 ^b^	13.8 ± 4.2
Creatinine (µmol/L)	14.0 ± 2.5	19.2 ± 5.2	16.5 ± 4.9	6.8 ± 5.8 ^c^

Every value includes the mean ± standard deviation. For each parameter, a superscript letter means that there is a statistical difference: ^a^ *p*-value of <0.0001 versus the Co, H+AL, and H+D2 groups; ^b^ *p*-value of <0.0001 versus the Co, H+CMC, and H+D2 groups; and ^c^ *p*-value of 0.0011 versus the H+CMC and H+AL groups. Co, control; H, hyperuricemia; CMC, sodium carboxymethylcellulose; AL, allopurinol.

## Data Availability

The raw data supporting the conclusions of this article will be made available by the authors on request.
